# Pulsed focused ultrasound can improve the anti-cancer effects of immune checkpoint inhibitors in murine pancreatic cancer

**DOI:** 10.1098/rsif.2021.0266

**Published:** 2021-07-07

**Authors:** Petros X. E. Mouratidis, Marcia Costa, Ian Rivens, Elizabeth E. Repasky, Gail ter Haar

**Affiliations:** ^1^ Joint Department of Physics, Division of Radiotherapy and Imaging, The Institute of Cancer Research—Royal Marsden Hospital, Sutton SM2 5NG, UK; ^2^ Department of Immunology, Roswell Park Comprehensive Cancer Centre, Buffalo, NY 14263, USA

**Keywords:** focused ultrasound, immunotherapy, pulsed high-intensity focused ultrasound, immune checkpoint inhibitors, pancreatic cancer

## Abstract

Pulsed high-intensity focused ultrasound (pHIFU) uses acoustic pressure to physically disrupt tumours. The aim of this study was to investigate whether pHIFU can be used in combination with immune checkpoint inhibitors (ICIs) to enhance survival of tumour-bearing animals. Murine orthotopic pancreatic KPC tumours were exposed both to a grid of pHIFU lesions (peak negative pressure = 17 MPa, frequency = 1.5 MHz, duty cycle = 1%, 1 pulse s^−1^, duration = 25 s) and to anti-CTLA-4/anti-PD-1 antibodies. Acoustic cavitation was detected using a weakly focused passive sensor. Tumour dimensions were measured with B-mode ultrasound before treatment and with callipers post-mortem. Immune cell subtypes were quantified with immunohistochemistry and flow cytometry. pHIFU treatment of pancreatic tumours resulted in detectable acoustic cavitation and increased infiltration of CD8^+^ T cells in the tumours of pHIFU and pHIFU + ICI-treated subjects compared with sham-exposed subjects. Survival of subjects treated with pHIFU + ICI was extended relative to both control untreated subjects and those treated with either pHIFU or ICI alone. Subjects treated with pHIFU + ICI had increased levels of CD8^+^IFNγ^+^ T cells, increased ratios of CD8^+^IFNγ^+^ to CD3^+^CD4^+^FoxP3^+^ and CD11b^+^Ly6G^+^ cells, and decreased CD11c^high^ cells in their tumours compared with controls. These results provide evidence that pHIFU combined with ICI may have potential for use in pancreatic cancer therapy.

## 1. Background

Pancreatic cancer is associated with poor prognosis and a high mortality rate. The majority of patients are diagnosed at an advanced stage of disease, too late for the surgery that is their only curative treatment option and that is only available to approximately 20% of patients [1]. Even after surgery, cancer will recur in 80% of patients, thus contributing to the dismal prognosis with 1, 5 and 10 year survival rates of 20%, 3% and 1%, respectively [2]. Chemotherapy and radiotherapy applied alone or in combination show little or no beneficial effect. Various facets of immunotherapy have been investigated clinically, including anti-CD40 antibodies [3], vaccines (e.g. GVax) [4], mesothelin-recognizing chimeric antigen receptor T cells [5] and oncolytic viruses (e.g. reolysin) [6], but no significant breakthroughs have yet been reported. The failure of treatments to provide therapeutic benefit has been attributed in part to the low number of neoantigens present in pancreatic tumours, a consequence of the relatively low number of mutations, and to a dense collagen-containing stroma [7], which creates a physical barrier around tumour cells, collapsing the vasculature [8] and shielding tumour cells from direct contact with T lymphocytes [9]. There thus remains a significant need for new therapeutic strategies to overcome these obstacles.

Advances in cancer immunotherapy have led to antibodies (immune checkpoint inhibitors (ICIs)) approved by the US Food and Drug Administration for the treatment of several cancers including melanoma and lung cancer [10–12]. ICIs include antibodies which target the Cytotoxic T lymphocyte antigen-4 (CTLA-4) and Programmed death-1 (PD-1) receptors expressed on the surface of cytotoxic T lymphocytes, and interfere with the function of regulatory signals which are naturally designed to inhibit, limit or reverse sustained activation of cytotoxic T lymphocytes [13,14]. Unfortunately, the therapeutic benefit of these treatments, which are designed to block the function of these naturally occurring inhibitory checkpoint receptors, has not been realized in pancreatic cancer patients. For example, a phase 2 clinical trial of patients with metastatic and locally advanced pancreatic cancer treated with Ipilimumab, which targets the CTLA-4 receptor, achieved a delayed clinical response in only 1 of the 27 patients treated [15]. Treatment modalities that can overcome the challenges that the dense physical barrier of the pancreatic tumour microenvironment poses to treatments could be of therapeutic benefit in an otherwise refractory disease when combined with immunotherapy. For example, preclinical evidence has shown that ultrasound treatments designed to cause thermal or mechanical damage to tumours can provide some anti-cancer benefit when combined with a number of immunotherapies including anti-CTLA-4 and/or anti-PD-1 antibodies, CD40 stimulation and toll-like receptor agonists [16–20].

High-intensity focused ultrasound (HIFU) is a physical modality that uses non-invasive focused acoustic beams [21] and that has been shown to enhance an anti-tumour immune response [22]. In HIFU's continuous ablative mode (exposures lasting a few seconds), the target focal temperature in tissue is greater than 55°C (often nearer to 80°C), resulting in cellular necrosis, but with some activation of programmed cell death pathways [23]. In pulsed mode (pHIFU) (exposures lasting milliseconds or less), which uses significantly higher pressure amplitudes, acoustic cavitation can occur. This may result in mechanical damage to the tissue in the absence of a significant temperature change [24]. Focused ultrasound has been used to treat pancreatic cancer either as a standalone treatment or in combination with chemotherapy and/or radiotherapy [25–28]. For example, a clinical review of 3022 patients with advanced pancreatic cancer showed that treatment with HIFU ablation alone, or in combination with chemotherapy, resulted in a median survival of 10 months, whereas treatment with chemotherapy alone yielded a median survival of 7.6 months. These treatments were well tolerated with no severe adverse effects [25] and show the feasibility of using HIFU in the clinic to treat pancreatic cancer. However, the use of HIFU in combination with ICI to provide anti-cancer benefit in pancreatic cancer has not as yet been investigated.

Some indication of the potential of combining pHIFU and ICI has come from eloquent studies in subcutaneous models of colorectal [17], neuroblastoma [18] and melanoma [19] tumours. An anti-vascular-related non-immunogenic mechanism was postulated for the improved tumour growth control seen in CT26 tumours when pHIFU (peak negative pressure (*P*−) = 1.65 MPa, frequency (*f*) = 1 MHz, 0.1 ms long pulses) was combined with microbubbles and anti-PD-1 antibodies [17]. By contrast, complete regression of tumour growth was seen in 62% of subjects when large (greater than 1200 mm^−3^) Neuro2A tumours were treated with higher rarefactional pressure exposures (*P*− = 14 MPa, *f* = 1.5 MHz, 13.3 ms long pulses) in combination with anti-CTLA-4/PD-1 antibodies and was associated with the induction of systemic immunity including the pro-immune regulation of natural killer cells, CD11c^+^ cells and circulating cytokines [18]. The release of neoantigens was demonstrated when B16F10 melanomas engineered to express the viral GP33 glycoprotein were treated with ‘histotripsy’ exposures (*P*− = 30 MPa, *f* = 1 MHz, 50 pulses) capable of improving tumour growth control in their own right [19]. Thus, it remains unclear whether the anti-cancer benefit of pHIFU + ICI treatments can be seen in deep-seated orthotopic pancreatic tumours where some attenuation of the ultrasound pressure wave can be expected, whether this effect can be associated with a traceable cavitation signal, and what the acute effects are of the pHIFU + ICI treatments on the balance of immune cell subsets in the tumours.

We predict that if pHIFU can mechanically disrupt tissue [29], then, if applied to dense tumours such as those of the pancreas, it could create an improved microenvironment allowing increased immune cell infiltration near to tumour cells, possibly increasing their efficacy if combined with ICIs, and lead to improved survival. This hypothesis is tested in the current study, in which the immunotherapy-refractory murine orthotopic pancreatic KPC tumour model was used to investigate whether pHIFU can induce mechanical damage in orthotopic pancreatic tumours to enhance the anti-cancer effects of anti-CTLA-4 and anti-PD-1 ICIs. To achieve this, we have overcome technical challenges associated with imaging and targeting of orthotopic pancreatic tumours, and provide cavitation data that show evidence of the induction of mechanical effects by the pHIFU treatments. The effects in the control and treatment groups on tumour burden and inflammation-associated biomarkers (tumour-infiltrating lymphocytes (TILs), cytokines, systemic immune cell subtypes) have been studied here. Evidence is provided that the combination of pHIFU and ICIs can produce improved anti-cancer effects relative to control subjects and to the treatments alone.

## 2. Methods

### 2.1. Cell lines and *in vivo* models

Murine pancreatic cancer KPC cells (*KrasG12D/+; Trp53R172H/+; Pdx-1-Cre*) supplied by Prof. Tuveson (Cold Spring Harbor Laboratory, USA) were used to establish syngeneic orthotopic tumours in C57BL/6J (000664) subjects [30]. KPC cells were maintained in a subconfluent monolayer at 37°C in 175 cm^2^ flasks in a humidified atmosphere containing 5% CO_2_. Screening for mycoplasma contamination was carried out before transplantation. Orthotopic KPC tumours were established by injecting 10^5^ KPC cells of low passage number (less than 3) in 10 µl of Matrigel into the pancreas (laparotomy) of immune-competent subjects (greater than 15 weeks old), as described in electronic supplementary material, S(a).

### 2.2. Treatment with pHIFU and ICI antibodies

**Figure 1.**
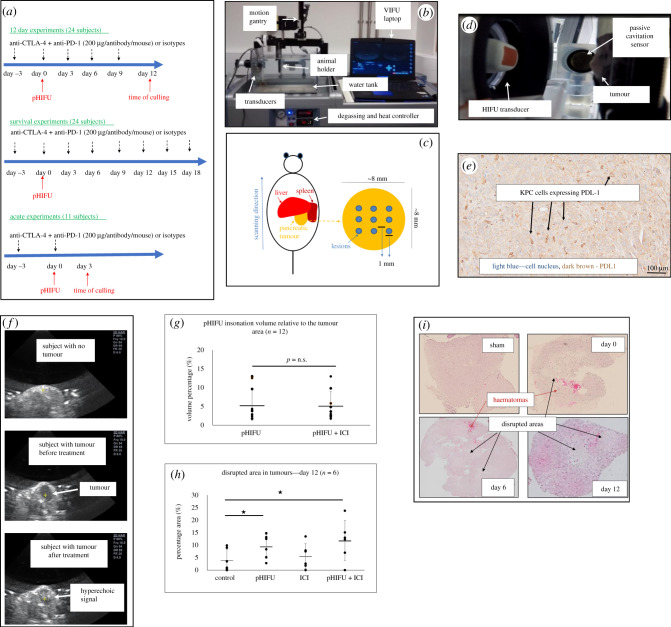
Characterization of baseline effects of pHIFU treatments on KPC tumours. (*a*) Treatment sequencing regime used in this study. Subjects were injected IP with a combination of anti-CTLA-4 and anti-PD-1 3 days before pHIFU exposures, on exposure day (immediately after pHIFU) and every 3 days thereafter until the time of culling (or up to 21 days for survival experiments). Subjects not treated with pHIFU were sham-exposed and subjects not treated with ICI were injected with isotype antibodies at the same dose and frequency as the ICI. (*b*) Alpinion small animal VIFU 2000 therapeutic ultrasound system comprising a 1.5 MHz HIFU transducer with a centrally mounted, co-aligned 7 MHz imaging probe for treatment guidance and monitoring facilitated by software control of the chosen target position within the mouse to the position of the focal peak using the automated gantry. (*c*) Schematic example representing targeting of the tumour with pHIFU. Lesions were placed every 1 mm in the horizontal and vertical direction. (*d*) Side-mounted broadband sensor used to detect cavitation (passive cavitation detector) in the HIFU focal volume. (*e*) PD-L1 expression in KPC tumours. (*f*) Typical examples of an image of a tumour-free animal, and a tumour imaged before and immediately after pHIFU with B-mode ultrasound are shown. (*g*) Comparison of the insonation volume of tumours in pHIFU and pHIFU + ICI subjects. (*h*) Percentage of tumour disrupted showing irregular or zero cell densities 12 days after treatment. (*i*) Staining of the tumours exposed to pHIFU + ICI and imaged immediately after and 6 and 12 days after treatment. Numerical results (*g*) and (*h*) are presented as means ± s.d. (*n* = 6) and statistical significance (denoted with an asterisk) is assumed at *p* < 0.05.

Subjects carrying orthotopic pancreatic tumours were randomly assigned to one of the four groups: (A) control group—sham-exposed to pHIFU to reveal any potential physiological effects of anaesthesia, handling and partial submersion in degassed water, and injected intraperitoneally (IP) with rat monoclonal IgG2A and mouse IgG2B isotype antibodies at the same concentration (200 µg/antibody/dose/mouse) and frequency (every 3 days) as the ICIs used in treatments, in order to mimic non-specific activation of the immune system (figure 1*a*; electronic supplementary material, table S1); (B) pHIFU treatment group—exposed to pHIFU using a small animal system (VIFU2000; Alpinion, USA) (electrical input power = 200 W, acoustic power = 132 W, peak positive pressure/*P*− = 32/17 MPa, pulse repetition frequency = 1 Hz, *f* = 1.5 MHz, duty cycle (d.c.) = 1%, exposure time = 25 s) (figure 1*b*; electronic supplementary material, table S2). In order to avoid damage to adjacent organs, only the central portion of the tumour was exposed. To exclude the possibility of wrongly identifying and targeting neighbouring tissues, the superior and inferior tumour edges were first identified under B-mode ultrasound imaging guidance. The tumours were then exposed to pHIFU under ultrasound guidance using a grid of horizontal and vertical lesions 2 mm apart (figure 1*c*). No lesion was placed within 2 mm of the tumour's superior or inferior edge or within 1 mm of its lateral edges. The number of pHIFU exposures for each tumour thus depended on the tumour dimensions and on the ability to differentiate tumour from neighbouring tissues. Subjects in this group were injected IP with the standard regime of isotype antibodies described above for the control group (figure 1*a*); (C) ICI treatment group—sham exposed to pHIFU and injected IP with the anti-CTLA-4/anti-PD-1 antibodies (200 µg/antibody/dose/mouse) (figure 1*a*; electronic supplementary material, table S1); and (D) combined pHIFU + ICI treatment group—treated with the pHIFU and anti-CTLA-4/anti-PD-1 antibodies as described above.

For tumour growth and immunophenotype analysis experiments (12 day experiments), 24 animals were used, 6 per experimental group. For survival experiments (up to 21 days), another 24 subjects were used (6 in each experimental group), with three separate experiments being carried out for each group. Eleven animals were used for acute experiments where immune phenotype analysis was carried out in tumours and lymphoid organs 48 h after treatment (figure 1*a*).

### 2.3. Treatment monitoring

Tumours were imaged in the cranial/caudal (sagittal) and medial/lateral (axial) imaging planes using two-dimensional high-frequency (14 MHz) B-mode ultrasound (E-Cube 9; Alpinion, USA). Images were recorded in planes 1 mm apart in order to obtain tumour dimensions in three orthogonal directions. At the time of culling, tumours were excised and their dimensions measured with Vernier callipers. For survival studies, subjects were culled when their tumour reached 300% of its initial volume (i.e. that measured 1 day before the pHIFU or sham exposure). Tumour volume was calculated assuming an ellipsoidal shape using

2.1 $$\mathrm{tumour}\;\mathrm{volume}\;({\mathrm{mm}}^{3})=\;\frac{\pi }{6}\times x\times y\times z.$$

### 2.4. Acoustic cavitation detection

Acoustic cavitation was monitored during pHIFU bursts using a weakly focused broadband (BB) polyvinylidine fluoride (0.1–20 MHz) passive cavitation detector (PCD) transducer (75 mm focal length, 15 mm active diameter; PA385; Precision Acoustics, UK). This was aligned with the pHIFU focal peak at the start of each treatment session using a pulse–echo technique (figure 1*d*). Cavitation data acquisition details have been published previously [31], and are also detailed in electronic supplementary material, S(b). The frequency range over which BB signals were integrated was 0.1–2.5 MHz. BB signals above reference levels (average off-time noise plus 3 s.d.) were assumed to provide a unique indicator of inertial cavitation [32]. In addition, the half-harmonic (HH) was quantified and compared with adjacent higher and lower reference frequencies. Data were processed using custom-written Matlab software (v. 7.0.1 R14; MathWorks Inc., USA) to obtain an HH and BB cavitation signal (in µV s) for each pHIFU exposure.

### 2.5. Immunohistochemistry

Excised tumours were formalin-fixed, paraffin-embedded and histologically stained with haematoxylin and eosin (H&E), Masson's trichrome stain and immunohistochemistry (IHC) antibodies. Details of these methods, the antibodies used and acquisition of images can be found in electronic supplementary material, S(c). To quantify this signal, ImageJ analysis software was used [33,34] for operator-independent automatic quantification, as has been performed in previously published studies [35–39]. Briefly, to determine the abundance of positively stained cells in whole tumour sections their composite images were created using Olympus's CellSens software at ×40 magnification. These were uploaded onto ImageJ and the brown (antibody stain) and blue (haematoxylin nuclear stain) colours were deconvolved using the IHC profiler function. The numbers of CD8^+^ and tumour cells were quantified automatically in whole tumour sections using the instructions and parameters shown in electronic supplementary material, tables S4 and S5. ImageJ was also used to determine the number of positively stained CD8^+^ cells in high-magnification tumour images and to provide their positional information in whole tumour sections as described in electronic supplementary material, S(d).

### 2.6. Flow cytometry

For analysis of immune biomarkers, the pancreaticoduodenal lymph nodes (TDLNs) draining the spleen and tumour were identified (electronic supplementary material, figure S1), excised and mechanically disintegrated to obtain single-cell suspensions through a 40 µm mesh filter. The cell fraction was collected after centrifugation for 5 min at 200*g*. To obtain a tumour cell fraction, tumours were cut into 3 mm pieces and enzymatically disintegrated for 1 h at 37°C using 0.2 Wunch units ml^−1^ of Liberase; the remaining intact tissue was further mechanically dissociated using a tissue grinder. Cells derived from tumour, spleen and TDLNs were counted with a haemocytometer and 10^5^ cells were transferred to flow cytometry tubes for staining. Cells were incubated with blocking buffer (phosphate-buffered saline (PBS)/1% fetal calf serum containing 1 : 100 dilution of anti-CD16/CD32 antibody (Abcam, UK)) for 1 h to block non-specific binding sites, and then incubated for 5 min with 4′,6-diamidino-2-phenylindole (DAPI) (3 µM) at 4°C to differentiate between live and dead cells. For staining of cell surface receptors, cells were immediately stained with primary antibodies conjugated to a fluorochrome for 1 h (1 : 100) at 4°C in the dark. For the staining of FoxP3, cells were fixed and permeabilized with the FoxP3 fixed and permeabilization buffer (Thermofisher Scientific Ltd) for 40 min. Then cells were washed twice with the permeabilization buffer provided in the kit and stained with (1 : 100 in permeabilization buffer) FoxP3 antibody for 1 h at room temperature. Then cells were washed twice with permeabilization buffer and then with PBS. Signal was detected immediately using a FACSCalibur LSRII (BD, USA). Murine live cells, T cells, cytotoxic T cells, regulatory T cells, myeloid-derived suppressor cells (MDSCs) and dendritic cells were defined as DAPI^−^, CD3^+^, CD3^+^CD8^+^, CD3^+^CD4^+^FoxP3^+^, CD11b^+^Ly6G^+^ and CD11c^+^, respectively [40].

### 2.7. Enzyme-linked immunosorbent assays

The Ready Set Go ELISA kits (E-bioscience/Affymetrix) were used to detect interferon-γ (IFNγ) and tumour necrosis factor-α (TNFα) in the blood plasma of subjects. Briefly, wells of a 96-well microwell plate were incubated overnight with IFNγ or TNFα biotin-conjugated antibody at 4°C. The next day the wells were washed five times with the wash buffer provided in the kit. Fifty microlitres of blood plasma/PBS mixture obtained from each subject was pipetted into each well. Alongside these wells, purified IFNγ and TNFα in increasing concentrations (0–10 ng ml^−1^) were added to quantify the test signal. After incubation for 2 h at room temperature, the supernatant was removed and plates were washed five times with the wash buffer provided in the kit. Plates were incubated with the capture antibody for 2 h at room temperature and then washed five times with the wash buffer. Plates were incubated with avidin–horseradish peroxidase for 30 min at room temperature, washed and then incubated with tetramethylbenzidine substrate solution for 15 min at room temperature. Stop solution was pipetted into the wells to end the development of the signal. Signal was assessed using a colorimetric plate reader at 405 nm with a reference wavelength at 495 nm.

### 2.8. Statistical analysis

Results are presented as Excel scatter column graphs of mean ± s.d. In all cases, *p*-values between individual groups were calculated using a one-tailed, two-sample unequal variance distribution with Student's *t*-test. Survival curves are presented as Kaplan–Meier plots using GraphPad Prism and a log-rank test for trend is used to identify statistically significant differences among the four groups in addition to Student's *t*-test. In all cases, *p*-values are considered significant at *p* < 0.05.

## 3. Results

### 3.1. Treatment of syngeneic KPC tumours with pHIFU and ICI

Our goal was first to characterize the baseline effects of pHIFU treatments on KPC tumours. Subject randomization and resulting initial group tumour volumes and dimensions for the survival and 12 day experiments are shown in table 1. Tumour dimensions at the time of treatment showed a variability of 1–3 mm. The variation in initial tumour volume among all animals was approximately 55%, with differences between treatment, sham and control groups not being statistically significant. IHC analysis of these tumours showed PD-L1 expression (figure 1*e*), and a collagen-containing microenvironment surrounding the tumour and the intercellular space (electronic supplementary material, figure S2). Tumours were visible under ultrasound imaging before and after pHIFU treatment (figure 1*f*). The insonated volumes in the pHIFU and the pHIFU + ICI treatment groups covered 5.3 ± 4% and 5.2 ± 3.8% of the whole tumour, respectively (figure 1*g*) and resulted in lesion-like (disrupted) areas which covered 9.5 ± 4.8% and 11.9 ± 8% of the whole tumour volume, respectively, 12 days after pHIFU treatment. These regions had exhibited lower (or zero) cell densities and were also seen in the subjects of the control and ICI groups (3.9 ± 4.8% and 5.6 ± 5%, respectively) possibly because of physiological necrosis of the tumour's core (figure 1*h*). The disruption of insonated tumours was evident immediately after pHIFU treatment. Haematomas were present, and disruption was sustained, as shown in pictures of tumours stained with H&E immediately, 6 and 12 days after treatment (figure 1*i*). We then investigated whether tissue disruption seen in pHIFU-treated subjects was associated with acoustic cavitation.

**Table 1.** Mean volumes and dimensions (*x, y, z*) of tumours at the beginning of their treatment.

	initial average volume (mm^3^)	initial average dimensions (*x*) (mm)	initial average dimensions (*y*) (mm)	initial average dimensions (*z*) (mm)
all groups (*n* = 48)	360 ± 190	9.3 ± 2.0	8.2 ± 2.1	8.2 ± 2.0
control (*n* = 12)	380 ± 240	9.3 ± 1.7	8.2 ± 1.8	8.1 ± 2.8
pHIFU (*n* = 12)	375 ± 210	9.8 ± 2.0	8.2 ± 2.4	7.3 ± 1.3
ICI (*n* = 12)	325 ± 110	8.9 ± 2.2	8.4 ± 2.5	8.3 ± 1.6
pHIFU + ICI (*n* = 12)	360 ± 170	9.0 ± 2.1	8.0 ± 1.9	8.8 ± 2.0

### 3.2. Acoustic cavitation detection

**Figure 2.**
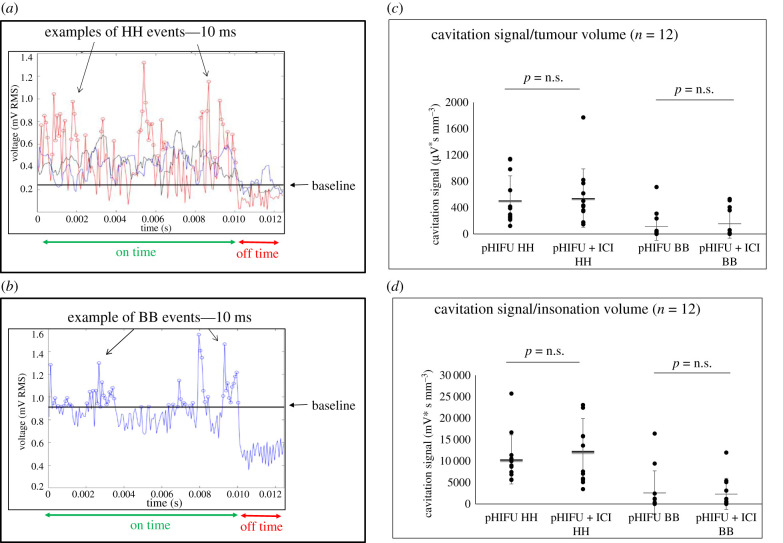
Detection of acoustic cavitation in KPC tumours. Sample dataset for cavitation detection during a 10 ms pHIFU pulse followed by an approximately 2 ms quiescent period. (*a*) 0.75 MHz half-harmonic (HH) signal as a function of time. This is indicative of stable bubble activity when in the absence of a broadband (BB) signal. HH events are shown as red points (circles). The horizontal black line (baseline) shows the average off-time noise signal (+ 3 s.d.) and the black and blue graphs show the reference frequencies signal (set as 1–3 bins below (blue) and above (black) HH). (*b*) BB emissions (indicative of highly energetic inertial cavitation activity, i.e. rapid bubble collapse) integrated over the range 0.1–2.5 MHz after software filtering to avoid drive harmonics and imaging signals as a function of time are shown. BB events are shown as blue points (circles). The horizontal black line (baseline) shows the average off-time noise signal (+3 s.d.). The HH and BB signals detected during pHIFU and pHIFU + ICI exposures are shown normalized for tumour volume (*c*), and insonation volume (*d*). Results are presented as means ± s.d. (*n* = 12) and statistical significance (denoted with an asterisk) is assumed at *p* < 0.05.

A representative example of the PCD signal obtained during a 10 ms HIFU pulse is shown in figure 2*a,b*. This shows continuously elevated HH emissions and frequently elevated BB signals (relative to the reference frequencies) that are additionally significantly above off-time levels. In addition, there was drive voltage kurtosis (e.g. around 2 and 4 ms) (electronic supplementary material, figure S3). Acoustic cavitation was seen in all subjects in the pHIFU and pHIFU + ICI groups. When normalized for tumour volume, subjects in the pHIFU and pHIFU + ICI groups showed HH signals of 510 ± 350 µV s mm^−3^ and 550 ± 400 µV s mm^−3^, respectively. Subjects in the pHIFU and pHIFU + ICI group showed BB signals of 120 ± 200 µV s mm^−3^ and 150 ± 200 µV s mm^−3^, respectively (figure 2*c*). These differences in the acoustic cavitation signal between the pHIFU and pHIFU + ICI groups were not statistically significant. Also, no statistically significant differences in HH and BB emissions/insonation volume were seen between pHIFU and pHIFU + ICI tumour treatments. Subjects treated with pHIFU, randomized into the pHIFU and pHIFU + ICI groups, showed HH signal/insonation volumes of 10 000 ± 6000 µV s mm^−3^ and 12 000 ± 7500 µV s mm^−3^, respectively, and BB signal/insonation volumes of 2600 ± 5000 µV s mm^−3^ and 2300 ± 3500 µV s mm^−3^, respectively (figure 2*d*). These results suggest that all pHIFU treatments induced acoustic cavitation, leading to mechanical disruption of tumours in both pHIFU and pHIFU + ICI groups. This led us to investigate whether survival could be increased using these treatments.

### 3.3. Survival and tumour growth results

**Figure 3.**
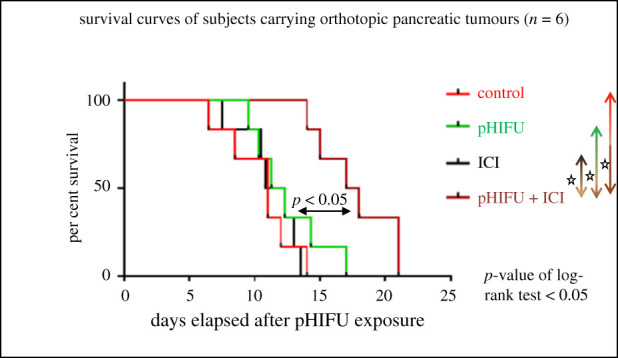
Survival of mice with orthotopic pancreatic KPC tumours after sham exposure or treatment with pHIFU and/or ICI. Subjects were culled when tumour volumes exceeded 300% of their initial volume. Results are presented as Kaplan–Meier plots, showing survival in days after the pHIFU exposure (*n* = 6). Statistical analysis with both a log-rank test for trend and a Student *t*-test shows that pHIFU + ICI-treated subjects had statistically significant improvements relative to all other treatments and control subjects. Statistical significance is assumed as *p* < 0.05.

Figure 3 shows improved survival for subjects treated with combined pHIFU + ICI with subjects surviving up to 21 days with a median survival of 17 days (range 13–21 days) after pHIFU treatments. Subjects in the control group survived for 10 days (range 6–14 days) only, subjects treated with pHIFU had a median survival of 12.5 days (range 10–17 days) and subjects treated with ICIs had a median survival of 11 days (range 8–14 days). These results showed a survival advantage in subjects exposed to pHIFU + ICI over all other groups, including the pHIFU group. To explain these results, the experiments were repeated and endpoints, including tumour growth and immune biomarkers, were assessed. Again subjects in the pHIFU + ICI group showed reduced tumour growth compared with that in tumours of subjects in the control, ICI and pHIFU groups 12 days after treatments (electronic supplementary material, figure S4).

### 3.4. Analysis of immune cell subsets in tumours

**Figure 4.**
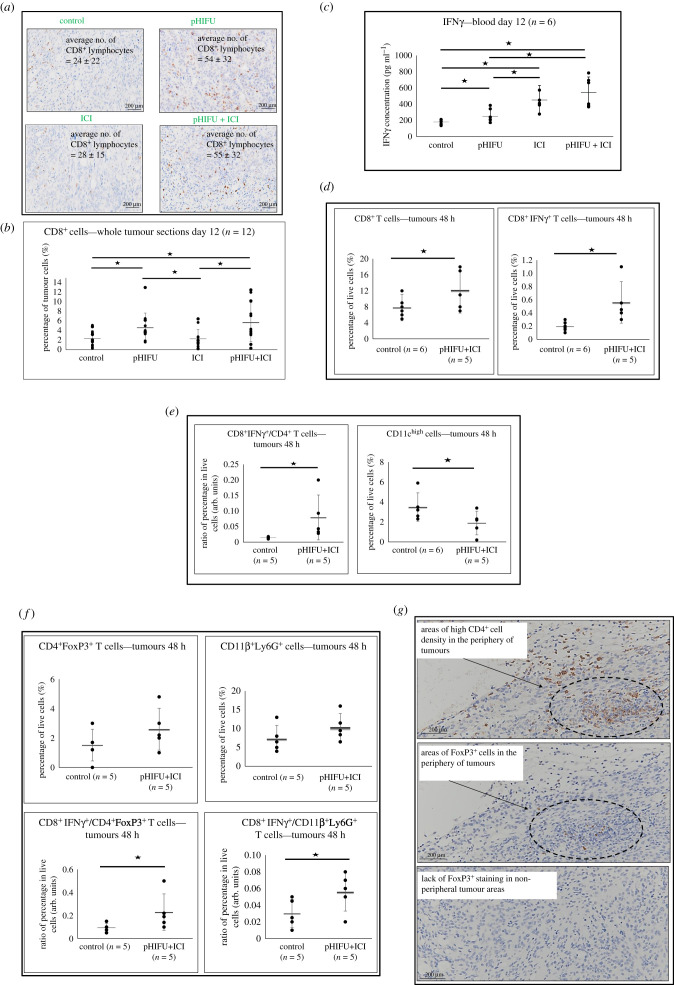
Immune cell analysis in tumours after pHIFU and ICI treatment. (*a*) Representative images of CD8^+^ tumour-infiltrating lymphocytes (stained brown) in orthotopic KPC pancreatic tumours excised from control, pHIFU, ICI and pHIFU + ICI-treated subjects 12 days after (sham) pHIFU. (*b*) Percentage ± s.d. (*n* = 12) of the number of CD8^+^ cells/the number of tumour cells in whole tumour sections of each control and treatment group (two whole tumour sections for each animal in every group were used to increase tumour coverage). (*c*) IFNγ concentrations in the blood of control, pHIFU, ICI and pHIFU + ICI-treated subjects 12 days after pHIFU treatment. Results are presented as means ± s.d. of six subjects. (*d*) Frequency of CD8^+^ T cells and CD8^+^IFNγ^+^ T cells in the tumours of control (*n* = 6) and pHIFU + ICI-treated (*n* = 5) subjects 48 h after treatment. Results are presented as the mean ± s.d. of live cell percentages. (*e*) Ratio of CD8^+^IFNγ^+^/CD4^+^ T cells and the frequency of cells expressing high levels of CD11c in control and pHIFU + ICI-treated tumours 48 h after treatment. Results are presented as the mean ± s.d. of percentages in live cells. (*f*) Abundance of CD4^+^FoxP3^+^ T cells and CD11b^+^Ly6G^+^ cells and their ratios to CD8^+^IFNγ^+^ T cells in the tumours of control (*n* = 5) and pHIFU + ICI-treated (*n* = 5) subjects. Results are presented as the mean ± s.d. (*g*) Examples of CD4^+^ and FoxP3^+^ cells in the periphery and non-peripheral areas of tumours as seen in control tumour sections. In all cases, statistical significance (denoted with an asterisk) is assumed at *p* < 0.05.

In order to investigate whether the increased cavitation signals, and the improved survival and tumour growth control of subjects in the pHIFU + ICI group were associated with increased infiltration of CD8^+^ lymphocytes in tumours, their abundance was assessed 2 and 12 days after treatment. Representative images of tumours of all subjects showed positive staining of CD8^+^ TILs 12 days after treatment. On average, images of control, pHIFU-treated, ICI-treated and pHIFU + ICI-treated tumours had 24 ± 22, 54 ± 32, 28 ± 15 and 55 ± 32 CD8^+^ cells per image, respectively (figure 4*a*). When whole tumour sections were assessed, a statistically significant increase in their abundance was detected in subjects in the pHIFU and pHIFU + ICI groups compared with those in control and ICI groups. In these tumour sections, the number of CD8^+^ T cells in the control, pHIFU, ICI and pHIFU + ICI groups (expressed as a percentage of the number of tumour cells) were 2.3 ± 1.6%, 4.7 ± 3.0%, 2.3 ± 1.9% and 5.8 ± 4.0%, respectively, 12 days after treatment (figure 4*b*). Furthermore, increased CD8^+^ TILs were seen within 0.5 mm of the tumour periphery compared with non-peripheral areas (0.5–5 mm from the tumour's edge) in ICI, pHIFU and pHIFU + ICI-treated subjects (electronic supplementary material, figure S5). A statistically significant increase in the levels of IFNγ, but not TNFα (electronic supplementary material, figure S6), was detected in the blood of subjects treated with pHIFU (250 ± 80 pg ml^−1^), ICI (450 ± 200 pg ml^−1^) and pHIFU + ICI (550 ± 180 pg ml^−1^) relative to that of controls (180 ± 20 pg ml^−1^) 12 days after treatment (figure 4*c*). Flow cytometric analysis of whole tumours 48 h after treatment with pHIFU + ICI showed increased levels of CD8^+^ and CD8^+^IFNγ^+^ T cells in these tumours relative to control subjects. Tumours of subjects treated with pHIFU + ICI showed that 12 ± 5% of their live (DAPI^−^) cells were CD3^+^CD8^+^, whereas this was 8 ± 3% in controls. Also, 0.6 ± 0.3% of all live cells in the tumours of pHIFU + ICI-treated subjects were CD8^+^IFNγ^+^ T cells, whereas this was only 0.2 ± 0.1% in controls (figure 4*d*). The CD8^+^IFNγ^+^/CD4^+^ T-cell ratio was increased in pHIFU + ICI-treated subjects (0.08 ± 0.04) compared with that in control subjects (0.01 ± 0.005). Also, 2 ± 1% of live cells in pHIFU + ICI-treated tumours were CD11c^high^, whereas this frequency was increased in control tumours (4 ± 1.5%) (figure 4*e*). In addition, non-statistically significant differences were seen in the frequency of CD4^+^FoxP3^+^ T cells and CD11b^+^Ly6G^+^ cells in the tumours of pHIFU + ICI-treated subjects compared with controls 48 h after treatment. The tumours of control and pHIFU + ICI-treated subjects showed that 2 ± 1% and 3 ± 1% of their live cells were CD3^+^CD4^+^FoxP3^+^, respectively, and 7 ± 4% and 10 ± 4% were CD11b^+^Ly6G^+^, respectively (figure 4*f*). An increase was detected in the ratios of CD8^+^IFNγ^+^/CD4^+^FoxP3^+^ T cells and CD8^+^IFNγ^+^/CD11b^+^Ly6G^+^ cells in pHIFU + ICI-treated tumours relative to controls. These ratios in control and pHIFU + ICI-treated subjects were 0.1 ± 0.05 and 0.25 ± 0.15 (CD8^+^IFNγ^+^/CD4^+^FoxP3^+^ T cells), respectively, and 0.03 ± 0.02 and 0.06 ± 0.02 (CD8^+^IFNγ^+^/CD11b^+^Ly6G^+^ cells), respectively (figure 4*f*). Also, 12 days after treatment, FoxP3^+^ cells were detected, irrespective of the treatment group, only in the periphery of the tumours (figure 4*g*). These results not only show the increased infiltration of CD8^+^ TILs into mechanically disrupted tumours, but also that the relative frequency of IFNγ^+^CD8^+^ T cells over that of other immune regulators including regulatory T cells and MDSCs increases within these tumours.

### 3.5. Analysis of immune cell subsets in the TDLNs and spleen

**Figure 5.**
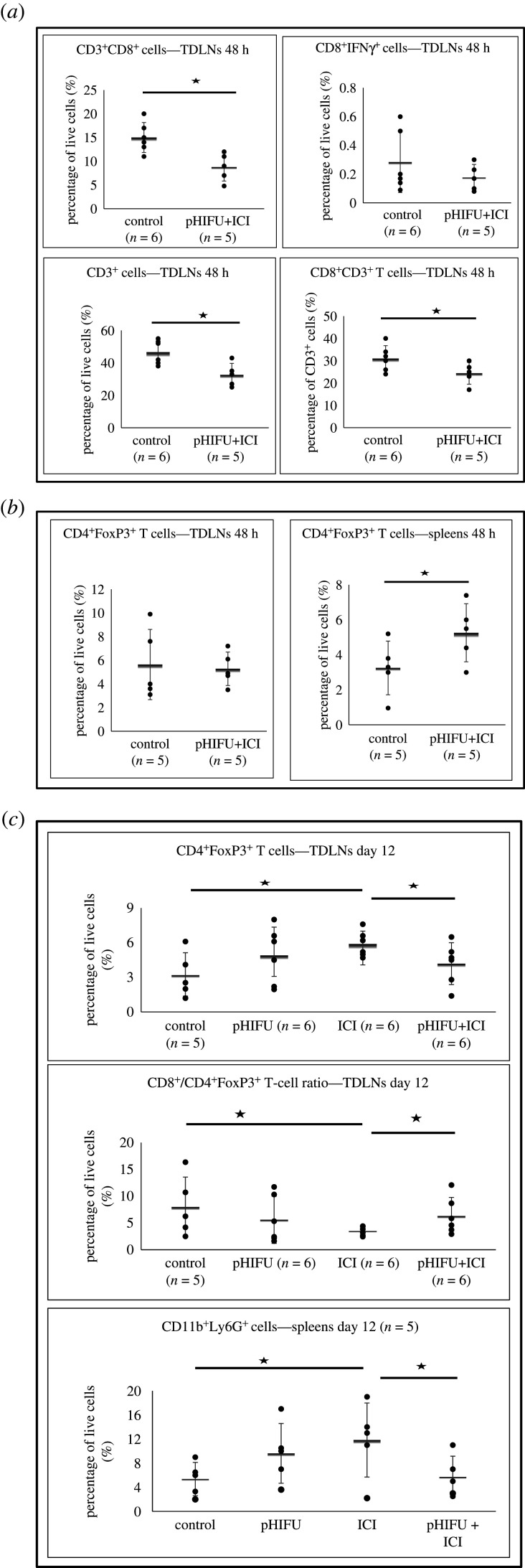
Immune cell analysis in the TDLNs and spleens after pHIFU and ICI treatment. (*a*) Percentage of CD8^+^ T cells, CD8^+^IFNγ^+^ T cells and CD3^+^ cells in the TDLNs of control (*n* = 6) and pHIFU + ICI-treated (*n* = 5) subjects and the frequency of CD8^+^ T cells in the CD3^+^ cell population 48 h after treatment. (*b*) Abundance of CD4^+^FoxP3^+^ T cells in the TDLNs and spleens of control (*n* = 5) and pHIFU + ICI-treated (*n* = 5) subjects 48 h after treatment. (*c*) Percentage of CD4^+^FoxP3^+^ T cells and their ratio to CD8^+^ T cells in the TDLNs and the frequency of CD11b^+^Ly6G^+^ in the spleens of control, pHIFU, ICI, pHIFU + ICI-treated subjects 12 days after treatment. In all cases, results are presented as the mean ± s.d. and statistical significance (denoted with an asterisk) is assumed at *p* < 0.05.

The decreased frequency of CD8^+^ T cells, but not that of CD8^+^IFNγ^+^ T cells, in the TDLNs of pHIFU + ICI-treated subjects relative to controls was observed 48 h after treatment. TDLNs of control and pHIFU + ICI-treated subjects showed that 15 ± 3% and 9 ± 3% of live cells were CD3^+^CD8^+^, respectively, and 0.3 ± 0.2% and 0.2 ± 0.1% were CD3^+^CD8^+^IFNγ^+^, respectively (figure 5*a*). The decrease in the abundance of CD8^+^ T cells in the TDLNs of pHIFU + ICI-treated subjects was associated with a decrease in CD3^+^ T cells in the TDLNs of pHIFU + ICI subjects and decreased frequency of the CD8^+^ T cells among the CD3^+^ cell population. The frequencies of these populations in control and pHIFU + ICI-treated subjects were 46 ± 7% and 33 ± 7% (CD3^+^ T/live cells), respectively, and 31 ± 6% and 24 ± 5% (CD8^+^/CD3^+^ T cells) 48 h after treatment (figure 5*a*). These results suggest that the acute effect of pHIFU + ICI treatments in the tumours is associated with the decreased draining of CD8^+^ T cells to the TDLNs of subjects.

A statistically significant increase was seen in the abundance of CD4^+^FoxP3^+^ T cells in the spleen but not in the TDLNs, of pHIFU + ICI-treated subjects compared with controls 48 h after treatment. Control and pHIFU + ICI-treated subjects had 6 ± 3% and 5 ± 1%, respectively (TDLNs), and 3 ± 1% and 5 ± 1%, respectively (spleens), of live cells that were CD3^+^CD4^+^FoxP3^+^ (figure 5*b*). No statistically significant differences were seen in the frequency of CD11b^+^Ly6G^+^ cells, nor in the ratios of CD8^+^/CD4^+^FoxP3^+^ T cells, CD8^+^/CD11b^+^Ly6G^+^, CD8^+^IFNγ^+^/CD4^+^FoxP3^+^ and CD8^+^IFNγ^+^/ CD11b^+^Ly6G^+^ in the spleens and TDLNs of control and pHIFU + ICI-treated subjects (electronic supplementary material, figure S7). To investigate whether the regulation of CD4^+^FoxP3^+^ T cells in the spleen and TDLNs is sustained longitudinally, their frequency was also investigated 12 days after treatment. Results showed a statistically significant increase in their frequency in the TDLNs of ICI-treated subjects compared with control and pHIFU + ICI-treated subjects (figure 5*c*), but not in their spleens (electronic supplementary material, figure S8). The percentage of these cells in the TDLNs of control, pHIFU, ICI and pHIFU + ICI-treated subjects were 3 ± 2%, 5 ± 2%, 6 ± 1% and 4 ± 2% of live cells, respectively, whereas the CD8^+^/ CD4^+^FoxP3^+^ T-cell ratios were 8 ± 5%, 6 ± 4%, 3 ± 1% and 6 ± 3%, respectively (figure 5*c*). In addition, ICI-treated subjects showed an increased frequency of CD11b^+^Ly6G^+^ cells (12 ± 6%) in their spleens relative to control and pHIFU + ICI-treated subjects (5 ± 3% and 6 ± 3%, respectively) 12 days after treatment (figure 5*c*).

## 4. Discussion

The aim of this study was to investigate whether combination treatments involving focused ultrasound and antibody immunotherapy might provide therapeutic potential for use in pancreatic cancer therapy. A murine orthotopic model which recapitulates some hallmarks of the human disease (K-RAS and p53 mutations [30] and collagenous structure (electronic supplementary material, figure S2)) was used to show that treatment of tumours with pHIFU + ICI resulted in (i) induction of acoustic cavitation and physical disruption of tumours, (ii) an improvement in cell survival and tumour growth control relative to that seen in control subjects and in those exposed to each treatment alone and (iii) the skewing of the balance of the immune contexture in the tumours towards a pro-immune phenotype by increasing the frequencies of CD8^+^ and CD8^+^IFNγ^+^ TILs, increasing the ratio of CD8^+^IFNγ^+^ T cells to CD4^+^ T cells, regulatory T cells and MDSCs, while controlling the frequency of the last two immune cell types in the tumours to basal levels.

In order to identify whether continuous HIFU or pHIFU exposure parameters are most likely to provide anti-cancer effects with immunotherapy, preliminary experiments showed that treatment with pHIFU resulted in increased CD8^+^ T cells in the blood of subjects relative to sham-exposed subjects 6 days after treatment, whereas continuous HIFU exposures (*P*− = 9 MPa, *f* = 1.5 MHz, *t* = 2 s, d.c. = 100%) did not (electronic supplementary material, figure S9). These data suggested that pHIFU exposures designed to induce mechanical damage in tissue in the absence of significant thermal change may be better equipped to create a pro-inflammatory environment than thermally ablative continuous HIFU treatments which are likely to heat fix target tissue, thus limiting intratumoral antigen release [16,19]. In order to facilitate clinical translation, we have used lower peak negative pressures and acoustic powers than those used in histotripsy studies [19,20]. Here ‘histotripsy-like’ pHIFU parameters (*P*− = 17 MPa, d.c. = 1%, 10 ms exposures) that were considered ‘safe’ were chosen to avoid the induction of non-specific inflammation since treatment with higher pulse negative pressures (*P*− ≥ 22 MPa), and attempts to ablate the whole tumour resulted in some skin and spleen damage. The dimensions of the focal volume of the ultrasound beam were 5 mm axially and 1 mm transversely. Therefore, to avoid damaging adjacent organs, tumours were treated when they were longer than 5 mm on axis. This resulted in a non-statistically significant variability in tumour dimensions and volume (table 1). This is not unreasonable, given the number of animals (48 subjects) used in survival and tumour growth studies. The tumour volumes at the time of culling were normalized to their initial values, thus allowing direct comparison of tumour growth and survival between different animals, and show that the results of this study are independent of tumour size.

All pHIFU exposures resulted in detectable acoustic cavitation. HH emissions, indicative of the activity of gas microbubbles inside the tumour which could disrupt and permeabilize the cells and tissues, were observed in all tumours. In addition, BB activity, suggestive of inertial cavitation, which could result in increased levels of destruction of the exposed volume, was detected in 10 of 12 tumours in each of the pHIFU and pHIFU + ICI groups. This is the only study on the effects of pHIFU and immunotherapy so far to document acoustic cavitation and thus verify the mechanical effect inside every treated tumour. This is of particular relevance in deep-seated orthotopic tumours where some attenuation of the focused ultrasound signal is to be expected. No statistically significant differences in cavitation emissions between pHIFU and pHIFU + ICI tumour treatments were seen (figure 2*c,d*), nor was there a difference between these two groups in the insonated tumour volumes (figure 1*g*). These results show that acoustic cavitation, suggestive of mechanical disruption and/or destruction of the cells and tissue, is induced in all pancreatic tumours treated with pHIFU and pHIFU + ICI, and that the anti-cancer benefit observed in pHIFU + ICI-treated subjects over the pHIFU-only treated subjects is not a consequence of technical variability during pHIFU exposures.

Subjects were treated with a PD-1 ICI to abrogate the immune inhibitory effects of the PD-1–PD-L1 axis since KPC cells were found to express PD-L1 (figure 1*e*). Anti-CTLA-4 was combined with anti-PD-1 as evidence suggests that the anti-CTLA-4 and anti-PD-1 blockades are conferred by different cellular mechanisms [41,42]. Our aim has been to maximize immunotherapy potency over the use of each antibody alone as anecdotal observations showed that this ICI combination, when given every 3 days, resulted in improved therapeutic efficiency relative to treatment with either antibody alone [43,44]. Using these ICI and pHIFU treatment parameters yielded a statistically significant increase in survival of approximately 40% relative to control subjects, approximately 25% relative to pHIFU subjects and approximately 35% relative to the ICI subjects for the combined pHIFU + ICI group (figure 3). By contrast, no statistically significant therapeutic benefit was seen for tumours treated with pHIFU or ICI alone compared with controls. This was reproduced in a second set of experiments where statistically significant improved tumour growth control for the pHIFU + ICI-treated subjects was seen, again compared with all other groups (electronic supplementary material, figure S4). Despite improved disease control, complete cure was not achieved, but this might be expected with such a difficult model where therapeutic options remain limited. This is a model with a high tumour burden and there is a short window from the moment the tumours are visible to that when they are of a size that can no longer be treated using our protocols. In some ways, this resembles the clinical situation where most pancreatic cancer patients die within months of diagnosis. The target of treatments in these cases is palliation and to increase the life of patients for as long as possible. The 40% improvement in survival seen in this study came with no observable side effects in the animal's quality of life (electronic supplementary material, table S7) [45], and if replicated in the clinic would lead to a significant improvement in disease prognosis. The aggressiveness of this tumour model also limited opportunities for the investigation of rechallenged tumours. Nevertheless, to the best of our knowledge, these remain the only results so far to show therapeutic benefit for the combined pHIFU + ICI treatment group relative to all other groups, in a deep-seated refractory orthotopic model like pancreatic cancer.

To provide a mechanistic basis for these results, we hypothesized that increased acoustic cavitation in the pHIFU + ICI-treated group could result in the enhanced infiltration of CD8^+^ TILs. To test this, TILs were investigated in tumours that were subjected to acoustic cavitation (pHIFU and pHIFU + ICI groups) and in tumours that were sham exposed (control and ICI groups) 12 days after treatment. IHC analysis was used instead of flow cytometry in order to avoid the extensive mechanical and enzymatic processing of relatively large and stiff pancreatic tumours (e.g. control group), and to provide positional information for the cells. No significant advantage of one technique over the other has been shown for biomarker analysis in previously published studies [46–48], and operator bias in the quantification of the abundance of stained cells on whole tumour sections was automatically minimized using ImageJ as previously reported [35–39]. Results showed that both pHIFU and pHIFU + ICI treatments approximately doubled the abundance of CD8^+^ T cells (figure 4*b*) relative to the control and ICI groups, suggesting that the cavitation induced by pHIFU allowed increased access to the tumours for these cells or created an environment which had a greater recruitment signal for antigen-activated T cells. However, expression of PD-L1 by the KPC cells (figure 1*e*) would be expected to limit their activation. To overcome this resistance, ICIs have been used, resulting in a therapeutic benefit in the pHIFU + ICI treatment group that was not seen in the pHIFU group (where no ICI were used). These results explain, at least partly, the beneficial anti-cancer effect seen in pHIFU + ICI subjects relative to all other treatment groups, and suggest that the mechanical disruption induced by pHIFU resulted in sustained elevated levels of CD8^+^ TILs in the tumours. When coupled with anti-PD-1 to abrogate the PD-1/PD-L1 inhibitory axis, and anti-CTLA-4 to maximize immunotherapeutic potency (e.g. by priming the immune system [49,50]), enhanced survival and tumour growth control was seen. In addition, analysis of the tumours, TDLNs and spleens of pHIFU + ICI-treated subjects revealed differential abundance of immune regulators compared with control subjects.

Tumour growth depends on the balance between pro- and anti-immune regulators [51]. Our results show that this balance within one of the most important tissues in the anti-cancer response, the tumour, was skewed towards a pro-inflammatory phenotype in pHIFU + ICI-treated subjects as early as 48 h after treatment. This was because these treatments caused the acute infiltration of CD8^+^ TILs and CD8^+^IFNγ^+^ TILs (figure 4*d*), raised the ratio of CD8^+^IFNγ^+^ TILs to regulatory T cells, MDSCs and CD4^+^ T cells (figure 4*e,f*) and retained the levels of regulatory T cells and MDSCs at the levels seen in control subjects (figure 4*f*). Also in murine, but not human, subjects, the reduction of cell surface CD11c staining (as seen here in pHIFU + ICI-treated tumours) (figure 4*e*) has been associated with the activation of dendritic cells [52]. These results suggest a change in the balance of the immune system inside the tumours towards a pro-inflammatory phenotype, and has not previously been observed at this early time point [18,19]. Finally, evidence from the TDLNs and spleens shows that pHIFU treatments decreased ICI-driven increases in negative regulators of the immune response, for example in T regulatory cells in the TDLNs and MDSCs in the spleen, thus further containing their anti-immune effect (figure 5*c*).

The data presented in this study give rise to questions the answers to which would provide further insights into the effects of the pHIFU and ICI combinations in pancreatic cancer. For example, a reduction in the frequency of CD8^+^ T cells seen in TDLNs (figure 5*a*) raises the question of whether this is due to the disruption of the tumour's lymph vessels by the ultrasound treatment. The disruption of the vasculature leading to decreased blood flow has been associated with the anti-cancer effects of pHIFU + anti-PD-1 [17]. Such mechanisms, which would regulate immune cell trafficking to the TDLNs, have not been investigated as yet. Second, increased levels of IFNγ in the blood of pHIFU + ICI-treated subjects compared with those in control subjects were seen (figure 4*c*), and the possibility that these could be indicative of an adverse systemic inflammatory response cannot be excluded. Cell depletion experiments (e.g. CD8 and/or IFNγ) would prove conclusively whether such a regulation exists. pHIFU-ICI-treated tumours show at least some irregular areas of collagen depletion and reorganization when compared with the dense-collagen microenvironment of control tumours (electronic supplementary material, figure S10). In order to design the most effective treatments, there is thus a need to increase our understanding of the effects of these treatments on the basic biological resistance mechanisms exploited by pancreatic ductal adenocarcinoma (PDAC). For example, differentiating between apoptotic (including immunogenic cell death) and acute necrotic events could show for how long the anti-cancer effects of the treatments could be sustained, and provide further explanation for the activation of the immune system. The use of repeated pHIFU treatments with the objective of augmenting the anti-tumour response using clinically feasible treatment parameters in genetically engineered PDAC subjects, and the application of modern high-throughput techniques (e.g. pan-transcriptome single-cell analysis) would aid those studies. In addition, longer endpoints would make possible the longitudinal characterization of the tumoral microenvironment, including the regulation of cancer-associated fibroblasts and macrophages [53] and a study of whether desmoplasia ensues or if debris is cleared.

## 5. Conclusion

A physical modality, focused ultrasound, has been used in combination with immunotherapy, in the form of ICI antibodies, to contain disease progression in KPC pancreatic tumour-bearing animals. A step-by-step justification for our pHIFU targeting decisions in order to mechanically disrupt these orthotopic tumours, without endangering the animal, has been given. The pHIFU-induced mechanical disruption of the tumours has been traced by detecting acoustic cavitation signals for each animal. Animals that were exposed to pHIFU + ICI survived longer than sham-exposed subjects or subjects treated with focused ultrasound or ICI alone. This improvement in disease progression in pHIFU + ICI subjects compared with control subjects has been explained by the increase of TILs in mechanically disrupted tumours, and the creation of a localized tumoral pro-inflammatory microenvironment when coupled with ICI. These results indicate that focused ultrasound may be able to potentiate the anti-cancer effects of traditional oncological interventions such as immunotherapy, and is a promising candidate for clinical use.

## Data accessibility

All data generated or analysed during this study are included in this published article and its electronic supplementary material. Data will also become publicly available in https://osf.io/collections/fusf/discover.

The data are provided in the electronic supplementary material [54].
